# Effects of a special continuous quality improvement in nursing on the management of adverse care events: a retrospective study

**DOI:** 10.1186/s12913-024-10913-4

**Published:** 2024-05-31

**Authors:** Qing Ouyang, Guixiang Zhang, Ying Xie, Hongman Yuan, Fangqun Cheng, Qiyun Huang

**Affiliations:** 1 Department of Nursing, The First People’s Hospital of Xiangtan City, No. 21 Shuyuan Road, Yuetang District, Xiangtan City, 411101 Hunan China; 2 Department of Nursing, Xiangtan Central Hospital, No.120 Heping Road, Yuhu District, Xiangtan City, 411100 Hunan China

**Keywords:** Nursing quality improvement, Adverse nursing events, Safety management

## Abstract

**Objective:**

To explore the application effect of the direct reporting system of adverse nursing events and special continuous nursing quality improvement measures in the management of these adverse events.

**Methods:**

The implementation time of continuous nursing improvement based on the direct reporting system was the demarcation point. We retrospectively collected and analyzed nursing adverse event reports and hospitalization data from Xiangtan Central Hospital before implementation (2015–2018) and after implementation (2019–2022). The active reporting rate of adverse events, the composition of these events and the processing time were compared between the two groups.

**Results:**

The rate of active reporting of adverse events before the implementation was lower than that after the implementation (6.7% vs. 8.1%, *X*^*2*^ = 25.561, *P* < 0.001). After the implementation of the direct reporting system for nursing events and the continuous improvement of nursing quality, the reporting proportion of first-level and second-level events decreased significantly. Moreover, the reporting proportion of third-level events increased significantly. The proportion of falls and medication errors decreased, and the proportion of unplanned extubation, infusion xerostomia and improper operation increased. The processing time of the reported nursing adverse events was significantly reduced (31.87 ± 7.83 vs. 56.87 ± 8.21, *t* = 18.73, *P* < 0.001).

**Conclusion:**

The direct reporting system of adverse nursing events and the continuous improvement measures for nursing quality can effectively improve the active reporting rate of adverse events, change their composition and reduce their processing time, as well as help create a safe psychological environment for both patients and nursing staff.

## Introduction

Patient safety is a paramount concern in healthcare, particularly within nursing management, where adverse events pose significant challenges [1]. Abnormal nursing accidents encompass a range of unplanned occurrences during patient care, such as falls, medication errors, and disorientation, which not only impact the reputation of healthcare institutions but also pose significant risks to patient well-being [2]. Studies indicate that a substantial proportion of medical mishaps, approximately 53%, occur within nursing practice [3]. Recognizing the critical importance of addressing these challenges, healthcare institutions strive to establish robust reporting systems to capture adverse events, facilitate learning from past errors, and enhance patient safety [4]. Moreover, the analysis and utilization of adverse event data serve as essential tools in identifying systemic issues within medical care processes, ultimately contributing to their prevention and the improvement of overall patient outcomes [4].

Adverse event reporting system collects a lot of data, which is very important in the management of adverse events. Ding Siqing et al. [5] summarized that scholars from all over the world used data mining technology to determine the high-risk factors and related factors of nursing adverse events such as medication, falls and stress injuries, and established a prediction model to take timely intervention measures, so as to prevent the occurrence of adverse events as soon as possible. As early as 2005, the World Health Organization proposed that all hospitals should have an adverse event reporting system, and it tends to be perfect, and also issued corresponding guidelines [6]. Many countries in the world have made efforts to establish and improve the adverse event reporting system. The nursing department at Xiangtan Central Hospital has implemented a direct reporting system for adverse events, alongside the establishment of a dedicated continuous nursing quality improvement group. These initiatives aim to proactively address patient safety concerns, optimize care delivery processes, and minimize the occurrence of adverse events.

The team conducts quality control and continuous improvement measures based on the adverse event report results of the direct reporting system. To evaluate the application effect of continuous nursing improvement based on the direct reporting system in the management of adverse events, the reported data on adverse events in the three years before and after implementation were retrospectively collected and analysed.

## Materials and methods

### General information

This research is based on a cross-sectional study, an observation of Xiangtan city central hospital, and a turning point in the implementation time of the system. The number of reported cases and the number of patients were collected and analyzed before implementation (2015–2018) and after implementation (2019–2022). Before we introduced the direct reporting system, data on adverse events were reported and collected through written report forms filled in by the parties concerned; generally speaking, grade III and IV events were submitted to the nursing department within 48 h, and discussion and analysis were completed within 7 days. Level I and II are reported by phone within 15 min, and discussion and analysis are completed within 48 h; adverse event reporting personnel include medical staff and nursing staff, and data management and exposure calculations are completed by full-time personnel; team members of the quality committee Members of clinical and functional departments from all majors and levels in the hospital participated, and the Quality Control Department took the lead in coordinating and completing the work with all medical staff.

General knowledge and consent for this study, and Xiangtan City Central Hospital University Review Committee, University Review No.: 2021-KY-28.

### Methods

The direct reporting system of adverse nursing events was established on the network nursing information platform on 1 January 2019. The direct reporting system was subsequently implemented, and the Nursing Safety and Quality Management Committee was established. Based on previous experience in the management of adverse nursing events, the Nursing Safety and Quality Management Committee established five continuous quality improvement teams for five specific aspects (falls, unplanned extubation, high-risk drug extravasation, drug-use errors and specimen collection errors). Continuous quality improvement teams typically consist of nursing specialists, healthcare quality experts, physicians, pharmacists, nurses, and data analysis experts to ensure that the team can comprehensively respond to adverse events and quality improvement needs. Each group includes a group leader, a supervisor and a secretary, as well as five group members. The work is divided among the team members, and each task is completed under the supervision of the team leader. The whole process is involved in the management, supervision and data archiving of specific adverse events. When adverse events are released in the system, discussion, analysis and continuous improvement must be carried out within the department and the hospital. When grade III and IV incidents occur in the department, the head nurse of the department will organize department members to discuss them. For grade I and II incidents, the nursing department will participate. Department event discussion meeting. The improvement of adverse nursing events follows the direction of the nursing department, supervision by the head nurse, and implementation by the nurses.

### Implementation of the direct reporting system for adverse nursing events

Through the direct reporting system of adverse events, real-time reporting and data summary analysis could be realised. Managers can dynamically monitor the occurrence of adverse nursing events and regularly conduct corresponding statistical analysis. The results of the data analysis can then be shared among all nursing staff through the nursing information platform, thus playing a role in supervision and vigilance.

The direct reporting system of adverse nursing events was officially implemented on 1 January 2019 [7]. The system includes the following aspects: (1) based on the principles of confidentiality, voluntariness and non-punishment, nurses are encouraged to compile reports on adverse events; (2) Nurses may report their own concerns anonymously or using their real names, and nurses may report concerns about other staff members (if they wish to remain anonymous, the identity of the reporter will be kept strictly confidential); (3) positive reports must be honest and based on personal experience, information should not be deliberately fabricated and others must not be slandered (if violations occur, relevant administrative and legal consequences will be borne according to the impact); (4) any violation of relevant laws and regulations in the report must not be subjectively intentional; (5) any initiative to eliminate medical and nursing safety risks must be given appropriate attention; (6) the collected adverse event reports and security risks are to be analysed monthly and made public on the website, Safety risks refer to things that are harmful to safety, that is, events that threaten the physical, life, and psychological safety of patients are called safety risks; and (7) the committee will evaluate the risk of adverse events, propose improvement measures for existing problems, continuously supervise rectification and evaluate the effects.

Adverse nursing events are defined as follows: any event that may affect the patient’s diagnosis and treatment results, increase their pain and burden, lead to medical disputes or medical accidents or affect normal operations and the personal safety of medical staff.

### Evaluation indicators

The active reporting rate of adverse nursing events was calculated as follows: nursing adverse events/total number of hospitalisations.

According to the medical safety (adverse) event reporting system of the Chinese Hospital Association, adverse nursing events are divided into four levels: Level I to Level IV. The lower the level, the more serious the consequences of the adverse event. For example, a Grade I event may result in minor injury or discomfort to the patient, while a Grade IV event typically results in death or serious long-term health effects. Medical institutions should take appropriate measures to prevent and handle adverse events based on the severity of the event [8, 9].

Other indicators included the classification of nursing adverse events and treatment time.

### Statistical analysis

The data were entered into EpiData 3.0 software (Odense, Denmark) through double entry. Subsequently, the data were converted into SPSS datasets and analyzed using the SPSS 22.0 statistical software package. The distribution of count data was described using percentages (%), and inter-group comparisons were conducted using the chi-square test (*X*^2^). For the distribution of measurement data, descriptive statistics such as mean ± standard deviation (x ± s) were utilized, and inter-group comparisons were performed using the independent sample t-test. Statistical significance was determined with a P-value of < 0.05.

## Results

### Active reporting rate and grading of adverse event reporting

There is little correlation between the gender and age of the two groups. Half of the demographic characteristics of the two groups of patients were comparable(*P* < 0.05). The results for the active reporting rate and grading of adverse event reporting between 2015 and 2022 are shown in Table 1. The rate of active reporting of adverse events was lower before the implementation (2015–2018) than after the implementation (2019–2022) (6.7% vs. 8.1%, *X*^*2*^ = 25.561, *P* < 0.001), indicating that the active reporting rate of adverse events had increased. There was a statistically significant difference in the composition ratio of adverse event classification before and after the implementation (*X*^*2*^ = 195.197, *P* < 0.001). After the implementation of the direct reporting system for nursing events and the continuous improvement of nursing quality, the proportion of first-level and second-level events reported decreased significantly. Moreover, the proportion of third-level events reported increased significantly (Table 1).

**Table 1:** Comparison of voluntary reporting rates of adverse events from 2015 to 2022

Years	Number of inpatient	Reports of adverse events (n, ‰)	Adverse event level (n, %)			
			Grade I	Grade II	Grade III	Grade IV
2015	41,823	211(5.0)	19(9.0)	58(27.5)	98(46.4)	36(17.1)
2016	42,341	203(4.8)	21(10.3)	45(22.2)	114(56.2)	23(11.3)
2017	47,812	357(7.5)	28(7.8)	56(15.7)	227(63.6)	46(12.9)
2018	44,382	414(9.3)	23(5.6)	41(9.9)	315(76.1)	35(8.5)
2015–2018	176,358	1185(6.7)	91(7.7)	200(16.9)	754(63.6)	140(11.8)
2019	46,811	413(8.8)	14(3.4)	20(4.8)	356(86.2)	23(5.6)
2020	52,172	350(6.7)	10(2.9)	23(6.6)	267(76.3)	50(14.3)
2021	51,681	456(8.8)	9 (2.0)	16(3.5)	389 (85.3)	42(9.2)
2022	57,213	471(8.2)	6(1.3)	14(3.0)	387(82.2)	64(13.5)
2019–2022	207,877	1690(8.1)*	39(2.3)	73(4.3)	1399(82.8)	179(10.6)

*Note* *There was a difference in the active reporting rate of adverse events before management (2015–2018) and after management (2019–2022) (X^2^ = 25.561, *P* < 0.001)

### Classification of adverse nursing events

The classification of adverse nursing events from 2015 to 2022 is shown in Table 2. After the implementation of the direct reporting system for nursing events and the continuous improvement of nursing quality, there was a statistically significant difference in the composition of nursing adverse events (*X*^*2*^ = 26.314, *P* < 0.001). The proportion of falls and medication errors decreased, whereas the proportion of unplanned extubation, infusion xerostomia and improper operation increased.

**Table 2:** Classification of nursing adverse event reports from 2015 to 2022

Years	Case	Adverse event classification (n, %)					
		Falls	Unplanned extubation	Infusion extravasation	Improper operation	Medication errors	Others
2015	211	80(37.9)	58(27.5)	42(19.9)	21(10.0)	6(2.8)	4(1.9)
2016	203	86(42.4)	53(26.1)	30(14.8)	13(6.4)	10(4.9)	11(5.4)
2017	357	91(25.5)	72(20.2)	58(16.2)	49(13.7)	41(11.5)	46(12.9)
2018	414	123(29.7)	89(21.5)	76(18.4)	56(13.5)	31(7.5)	39(9.4)
2015–2018	1185	380(32.1)	272(23.0)	206(17.4)	139(11.7)	88(7.4)	100(8.4)
2019	413	124(30.0)	99(24.0)	97(23.5)	47(11.4)	20(4.8)	26(6.3)
2020	350	107(30.6)	90(25.7)	78(22.3)	35(10.0)	22(6.3)	18(5.1)
2021	456	156(34.2)	98(21.5)	103(22.6)	54(11.8)	20(4.4)	25(5.5)
2022	471	106(22.5)	130(27.6)	110(23.4)	70(14.9)	20(4.2)	35(7.4)
2019–2022	1690	493(29.2)*	417(24.7)	388(23.0)	206(12.2)	82(4.9)	104(6.2)

*Note* *There was a difference in the Adverse event classification before management (2015–2018) and after management (2019–2022) (X^2^ = 26.314, *P* < 0.001)

### Adverse nursing event processing time

The processing time of adverse nursing events from 2015 to 2022 is shown in Table 3. After the implementation of the direct reporting system of nursing adverse events, combined with the implementation of the continuous improvement of nursing quality team, the processing time of the reported nursing adverse events significantly decreased (31.87 ± 7.83 min vs. 56.87 ± 8.21 min, *t* = 18.73, *P* < 0.001).

**Table 3:** Comparison of treatment time for voluntary reporting of nursing adverse events from 2015 to 2022(min)

Years	Number of cases	Duration (minutes)^*^
2015	211	59.17 ± 8.33
2016	203	58.98 ± 7.86
2017	357	59.13 ± 7.80
2018	414	48.00 ± 7.50
2015 ~ 2018	1185	56.87 ± 8.21
2019	413	33.73 ± 6.95
2020	350	30.17 ± 7.27
2021	456	31.68 ± 6.87
2022	471	31.19 ± 5.78
2019 ~ 2022	1690	31.87 ± 7.83*

*Duration (minutes): From 2015 to 2022, the time limit for reporting malpractice incidents is the time limit. *There was a difference in the treatment time of adverse events before management (2015–2018) and after management (2019–2022) (X^2^ = 18.73, *P* < 0.001)

## Discussion

### Establishment of a direct reporting system for adverse nursing events and the clarification of the non-punishment and active reporting system

The results of this study showed that the total number of reported adverse events increased following the implementation of the system and that the difference was statistically significant (*P* < 0.05), which indicated that the direct reporting system combined with the implementation of the continuous improvement of nursing quality team could effectively increase the reporting rate of adverse events. The liability-free direct reporting system and the continuous improvement team have the characteristics of direct reporting, supervision and protection, which are of great significance to encouraging the reporting of adverse nursing events and ensuring the quality and safety of nursing [10–12]. Yang Yuxiu et al. [13] demonstrated that the factors that affect the reporting of adverse nursing events include lack of confidentiality, the complexity of the reporting process and the lack of a fully realised non-punitive system. Kuang Zixia [14] noted that incidents that threaten patient safety are often caused by problems in the multiple linkages within the system, such as medication errors and delays in treatment, often stem from problems in multiple linkages within the healthcare system, including communication breakdowns, workflow inefficiencies, equipment failures, staffing shortages, and inadequate protocols. It is necessary to establish a non-punitive automatic reporting system for error-related events and a medical safety (adverse) event management model based on this.

In the present study, a direct reporting system for adverse nursing events was established, with the punitive culture abandoned and a relaxed and harmonious atmosphere created in the department, wherein the medical staff were encouraged to take the initiative in reporting various near-misses in their work and valuable information and certain economic incentives were provided, such as financial rewards or bonuses, to create a non-punitive management environment. The Nursing Quality and Safety Committee analysed the reasons behind reporting errors and accidents, learned from previous mistakes and drew up lessons to have a more comprehensive grasp of patient safety information and to subsequently analyse, improve and adjust it accordingly. Each clinical department should pay more attention to the hidden dangers pertaining to nursing safety through the regular summary and analysis of adverse events and the rectification proposals given by the continuous nursing quality improvement group [15–17]. According to the results of this study, the establishment of a direct reporting system for adverse events and a clear non-punitive and active reporting system can effectively improve the motivation of nurses to actively report such events and increase the number of reported incidences.

### Enhancing nursing quality and safety

The results of this study show that following the implementation of quality management by the continuous improvement team, the proportion of grade I and grade II events decreased significantly, whereas the proportion of grade III and grade IV events increased significantly, with the difference statistically significant (*P* < 0.05). This indicated that the implementation of this system had a positive effect on the rectification of adverse events. Xiang Q [18] noted that while adverse nursing events cannot be completely avoided, they can be reduced or partially avoided through effective risk-event management. The core element of this management is ensuring the safety of patients and evaluating the quality of the nurses’ work process. Furthermore, nursing quality entails a process of continuous improvement. Using a good quality management mechanism can help evaluate the nursing quality and subsequently improve it. The overseas scholars, John and Sigmon, also affirmed the role of good nursing management tools in ensuring nursing quality and safety [19, 20], such as nursing quality risk management early warning system, while according to other reports [21, 22], nursing quality control presents a purposeful management behaviour. Effective control depends on two factors, the first of which relates to having a clear purpose, that is, there must be clear standards for quality of care, while the second factor relates to the existence of corresponding means to achieve this purpose, such as human, material, financial, informational and organisational structure means, which serve as the basis for achieving quality-related goals. The domestic scholars, Ma Ling and Wu Fengping, carried out effective nursing quality control based on the above aspects and achieved good results [23, 24].

In the process of the continuous improvement of nursing quality, this study clarified the nursing quality standards and devised a nursing quality framework to set up a management committee aimed at ensuring the achievement of the quality-related goals. The implementation of the continuous improvement of nursing quality can help reduce the occurrence of adverse events and improve both the quality and the safety.

The results of this study indicated that the rate of active reporting of nursing adverse events was higher following the implementation of the system than before the implementation. With the implementation of the direct reporting system of nursing incidents and the continuous improvement of nursing quality, the proportion of first- and second-level incident reports has significantly decreased, and the proportion of third-level incident reports has increased significantly. For example, the proportion of falling from bed decreased from 40.3 to 37.5%, and the proportion of drug-use errors decreased from 7.4 to 4.9%. Furthermore, various types of adverse events were reported, including the patient going out privately, burns, suicide, nurse injuries and inadequate teaching of interns. This showed that the nursing staff had changed their understanding of adverse events. Specifically, the content of the adverse event reports included not only errors or accidents that must be reported but also critical events involving potential safety hazards. Adverse events pertaining to nursing deficiencies that were not taken seriously were also reported.

### Reducing the processing time of adverse events and improving the quality of care

The committee classified and summarised all the reported adverse events and sent the results to the continuous improvement team. The members of each group subsequently conducted an in-depth analysis of the adverse event data, proposed effective rectification measures for the causes and implemented them to effectively prevent the re-occurrence of the adverse events, for example, additional staff training on fall prevention techniques, revision of medication administration protocols, implementation of standardized procedures for intravenous infusion, enhancement of nursing protocols for specific patient populations, and adoption of technology solutions to support patient safety initiatives. These proactive measures taken by the continuous improvement team aim to address identified issues and mitigate potential risks, thereby enhancing patient safety and nursing care quality. In the process of analysing the causes, it was found that certain adverse events were caused by poor work systems or processes. Therefore, the committee revised the working system and optimised the workflow at the hospital or department level, which included establishing standards such as the ‘standardised assessment process for high-risk fall patients’, ‘ten-step method of intravenous infusion’ and ‘standardised procedures for intravenous treatment and nursing’. The continuous improvement team also adopted preventive measures in a planned and targeted manner to strengthen the monitoring and management of key links and key populations. Key links refer to critical steps or processes within a system that have a significant impact on its overall performance or outcomes. These may include crucial points in workflow, communication channels, or decision-making processes that are essential for achieving desired results. Key populations typically refer to specific groups of individuals or patients who are at higher risk or have particular needs within a healthcare context. These populations may include patients with certain medical conditions, elderly patients, pediatric patients, or individuals from vulnerable or marginalized communities. Identifying and addressing the needs of these key populations is important for providing targeted and effective healthcare interventions. Through scientific analysis, timely feedback and rapid rectification of reported events, the processing time of adverse events was significantly reduced. This showed that the continuous improvement group could effectively ensure the safety of patients and nursing staff. The continuous improvement group also focused on the timeliness of event discovery, the effect of event intervention and the improvement of the hospital management system. Compared with traditional medical quality management, this system demonstrates higher work efficiency and a more complete monitoring range.

During this study some limitations needs to be considered when interpreting the findings. At the beginning of this research, there are problems with the number of repetitions and collection methods, which may lead to deviations or incomplete information. Secondly, the research relies on self-reporting of delinquent cases, possible existence of insufficient reporting, or circumstances in which the classification of cases is not certain. In addition, the characteristics of the collection of numbers, the reporting of the possibility of existence selection or the lack of truth, and the possibility of the outcome of the research. In addition, the scope of this research is limited to the specific medical equipment and practical environment, which limits the possible reach of the research results. Finally, we have completed the implementation of a series of measures and restraint potential biases, but the possible existence and other factors and effects of complete restraint have been researched. Because of this, when we study the results of our research, we consider the demand carefully and consider its small locality, so we continue to explore the future in a comprehensive manner.

## Conclusion

The direct reporting system of adverse nursing events and the continuous improvement team for nursing quality cover the whole process of nursing quality and safety management. The system can effectively improve the active reporting rate of adverse nursing events, change their composition and reduce their processing time, as well as create a safe cultural environment for both patients and nursing staff.

## Implication

In the process of continuous improvement of nursing quality, this study clarified the nursing quality standards, and organized a nursing quality framework to set up a nursing management committee to ensure the completion of nursing quality goals.

This study shows that the implementation of continuous improvement of nursing quality can reduce the occurrence level of nursing adverse events and improve the quality and safety of nursing.

## Data Availability

All data generated or analysed during this study are included in this published article.
